# Expulsion of Tænia by Copper

**Published:** 1861-11

**Authors:** 


*Expulsion of Tænia by Copper.*—Dr. Thienemann, of Prussia, says that he has invariably succeeded in expelling tape-worm by oxide of copper, administered in one-grain doses four times a day. The doses, he says, may be much increased without bad effect.—*Medical and Surgical Reporter.*

